# The evaluation of oxidative stress parameters in breast and colon cancer

**DOI:** 10.1097/MD.0000000000025104

**Published:** 2021-03-19

**Authors:** Berrin Papila Kundaktepe, Volkan Sozer, Sinem Durmus, Pinar Cigdem Kocael, Fatih Orkun Kundaktepe, Cigdem Papila, Remise Gelisgen, Hafize Uzun

**Affiliations:** a Department of General Surgery, Faculty of Cerrahpasa Medicine, Istanbul University-Cerrahpasa; b Department of Biochemistry, Yildiz Technical University; c Department of Medical Biochemistry, Faculty of Cerrahpasa Medicine, Istanbul University-Cerrahpasa; d Department of Internal Medicine, Taksim Research and Training Hospital; e Department of Internal Medicine, Division of Oncology, Faculty of Cerrahpasa Medicine, Istanbul University-Cerrahpasa, Istanbul, Turkey.

**Keywords:** advanced protein oxidation products, breast cancer, colon cancer, ischemia modified albumin, malondialdehyde, nitric oxide, prooxidant-antioxidant balance, total antioxidant capacity

## Abstract

Our aim in this study was to investigate the relationship between serum ischemia modified albumin (IMA) levels with oxidative stress parameters [protein carbonyl (PCO), advanced protein oxidation products (AOPPs), malondialdehyde (MDA), total nitric oxide (NOx), prooxidant-antioxidant balance (PAB), and ferric reducing of antioxidant power (FRAP)] in breast cancer (BC) and colon cancer (CC).

In total, 90 patients undergoing surgical treatment for BC (n = 45) or CC (n = 45) and 35 healthy controls were included in this cross-sectional study.

The serum PCO, AOPPs, MDA, NOx, PAB, and IMA levels were all statistically significantly higher in the cancer patients than in the control group. MDA, NOx, and PAB levels were significantly lower in the BC group than in the CC group. FRAP values were statistically significantly lower in both the CC group and the BC group compared to the control. IMA showed a weak positive correlation with CA-19.9 (*r* = 0.423 *P* = .007) but a moderate positive correlation with tumor size in the CC group. IMA showed a positive correlation with metastasis, grade, and HER2 and a negative correlation with ER and PR in the BC group.

Oxidative stress is a key player in the development of solid malignancies. Cancer development is a multistage process, and oxidative stress caused by the production of ROS/RNS in the breast and colon may predispose individuals to BC and CC. Patients with BC and CC had an impaired oxidative/antioxidant condition that favored oxidative stress. The ROC analysis indicated that IMA sensitivity above 80% could be used as a secondary biomarker in diagnosis.

## 1 Introduction

Breast cancer (BC) and colon cancer (CC) are among the most common cancer types in the world and are the most frequent cause of death due to cancer. One report in 2020 concluded that BC was the most common type of cancer diagnosed in women, as it is seen in 1 of every 4 women with cancer.^[1]^ By contrast, CC ranks 4th as the most frequently diagnosed cancer type and 2nd in cancer-related deaths, affecting women and men at almost the same rate.^[2,3]^ The 5-year relative survival rates are 64.6% for CC and 90% for BC, but survival depends strongly on the stage of disease at diagnosis. Typically, the 5-year survival rate ranges from 90.2% for CC and 63% for BC when detected at the localized stage, but this declines to 71.8% (CC) and 30% (BC) for regional disease and to 14.3% (CC) and 6% (BC) for distant metastatic cancer.^[4]^ In addition, approximately 30% of patients with early-stage BC have recurrent disease, most of which is metastatic.^[5]^ For these reasons, the ability to estimate the pre-treatment prognosis of patients with CC or with BC is valuable in assessing the patient's future condition and quality of life. Therefore, much effort has been expended in searching for screening, prognostic, and predictive biomarkers for patients with BC and CC.

Many types of cancer show a persistent increase in oxidative stress due to a reduced effectiveness of the antioxidant system or to an increased production of reactive oxygen species (ROS) and nitric oxide (NO)/reactive nitrogen species (RNS). The effects of increased ROS and RNS vary according to their radical forms, concentrations, and where they occur,^[6]^ but they affect cancer cells by triggering DNA damage, stimulating genetic mutations, and inhibiting apoptosis, proliferation, invasion, and metastasis. Therefore, the antioxidant/oxidative parameters of the tumor are prognostically important in many types of cancer, and these parameters can be assessed by the detection of well-known oxidative markers of proteins, such as protein carbonyl (PCO), advanced oxidation protein products (AOPP), and ischemia modified albumin (IMA),^[7,8]^ or of malondialdehyde (MDA), a lipid peroxidation marker.^[9]^ For example, serum IMA levels are increased in various cancers, including BC and CC.^[8,10–16]^

One assay for determining the antioxidant/oxidative parameters is the prooxidant-antioxidant balance (PAB) technique, a new strategy that determines the prooxidant load and antioxidant capacity in a single assay.^[17]^ The PAB assay provides general view of the oxidant/antioxidant status of the patients in 1 single experiment. Similarly, the total antioxidant capacity (TAC) can be measured as the ferric reducing of antioxidant power (FRAP), which determines the capacity for reduction of Fe^3+^ (ferric ion) to Fe^2+^ (ferrous ion) in the presence of antioxidants.^[18]^ This ability to measure the antioxidant/oxidative parameters also provides the ability to use these parameters as prognostic markers. However, not many studies have evaluated these parameters as markers in BC and CC.

## 2 Objective

In this context, our aim in this study was to examine the levels of oxidant and antioxidant molecules in CC and BC and to determine the effectiveness of these measurements in distinguishing metastatic/high grade disease and high-stage patients.

## 3 Materials and methods

This study was conducted at the Department of Internal Medicine, Division of Oncology, and Department of General Surgery, Faculty of Cerrahpasa Medicine, Istanbul University-Cerrahpasa. The protocol for sample collection was approved by the Ethical Committee (number: 83045809/604.01/04-47792) of the Cerrahpasa Medical Faculty. The study was performed in accordance with the tenets of the Helsinki Declaration, and informed consent was obtained from all patients and controls prior to their inclusion in the study.

A total of 90 patients were admitted during the study period. Thirty five criterion-matched healthy individuals were also enrolled into this study. Exclusion criteria in both the study and control groups included cardiovascular diseases, diabetes mellitus, renal failure, autoimmune disease, chronic infection and inflammation, alcohol abuse, or use of antilipidemic, antioxidant, anti-inflammatory, corticosteroid, or immunosuppressive drugs. All participants also answered survey questions on demographics, diet, and lifestyle, and those with similar lifestyle and diet were included in the study. Any patients with neoadjuvant treatment were also excluded from study. Ultimately, the study group included 45 patients with BC and 45 patients with CC. None of the included patients had both breast cancer and colon cancer.

The patients with BC in this study had distant metastases at the time of diagnosis. We evaluated their clinicopathological features (histology, menopausal status, estrogen receptor (ER), progesterone receptor (PR) status, number of axillary lymph nodes involved, grade, and tumor size and stage according to the American Joint Committee on Cancer staging system. The patients with CC had newly diagnosed and histologically confirmed primary colorectal cancer, and their tumors were staged according to the Dukes’ and TNM Classification of Malignant Tumors (TNM).

Blood was drawn from both groups in the morning after 12 to 14 hours of fasting. Serum was obtained, after at least 30 minutes of clotting, by centrifugation at 2500 g for 15 minutes. Part of the serum was used directly for measurements of biochemical parameters and tumor markers, and the remainder was stored at −80°C until assayed for determination of the other parameters. Any icteric or hemolytic blood samples were discarded. All parameters were analyzed in all samples together in a single batch, after completion of our protocol (control and patient samples were analyzed in the same batch).

### 3.1 Measurement of serum PCO concentrations

PCO levels were measured by the method of Reznick and Packer,^[19]^ with slight changes to allow working with small volumes of serum. The coefficients of the intra- and inter-assay variation were 4.9% (n = 20) and 5.7% (n = 20), respectively.

### 3.2 Measurement of malondialdehyde (MDA) levels

Lipid peroxidation status was ascertained by the formation of MDA as an end product of fatty acid peroxidation. MDA levels were measured in plasma using the methodology of Buege and Aust,^[20]^ which is based on the measurement of the color produced during the reaction between thiobarbituric acid and MDA by spectrometry at 535 nm. The coefficients of intra- and inter-assay variation were 4.2% (n = 20) and 4.9% (n = 20), respectively.

### 3.3 Measurement of serum advanced oxidation protein products (AOPP)

The levels of AOPP were measured using the method of Hanasand,^[21]^ with slight modifications to allow working with small amounts of serum. The coefficients of intra- and inter-assay variation were 5.1% (n = 20) and 6.1% (n = 20), respectively.

### 3.4 Measurement of serum total nitric oxide (NOx) concentrations

Serum NOx was measured using an enzyme-linked immunosorbent assay (ELISA) kit (SinoGeneClon Biotech Co., Ltd, Hangzhou, China), as per the manufacturer's instructions. The coefficients of intra- and inter-assay variation were 4.2% (n = 20) and 5.3% (n = 20), respectively.

### 3.5 Measurement of serum ischemic modified albumin (IMA) levels

Serum IMA levels were measured in duplicate aliquots, using a human ELISA kit in accordance with the manufacturer's instructions (Eastbiopharm Co. Ltd., HANNGZHOU). The coefficients of intra- and inter-assay variation were 5.2% (n = 20) and 6.2% (n = 20), respectively.

### 3.6 Measurement of the serum prooxidant-antioxidant balance (PAB)

The PAB was measured with the method of Alamdari et al,^[17]^ with slight modifications. The oxidation-reduction indicators used in this method were 3,3’,5,5’-tetramethylbenzidine (TMB) and TMB cations, which have different optical and electrochemical properties. The coefficients of intra- and inter-assay variation were 5.1% (n = 20) and 6.0% (n = 20), respectively.

### 3.7 Measurement of serum ferric reducing antioxidant power (FRAP)

The antioxidant status of the serum samples was measured with the FRAP assay, which is a redox-linked colorimetric method that uses reductant antioxidants.^[18]^ The coefficients of intra- and inter-assay variation were 5.1% (n = 20) and 6.3% (n = 20), respectively.

Biochemical parameters were measured by enzymatic methods using commercial kits (Roche Diagnostics, GmbH, Mannheim) with an Olympus AU 800 analyzer located in the Central Biochemistry Laboratory of Cerrahpasa Medical Faculty. Tumor markers were measured by immunometric assays on an IMMULITE 2000 immunoassay analyzer (DPC, Los Angeles, CA).

### 3.8 Statistical analysis

All statistical analyses were carried out using SPSS v. 22.0 (IBM, Armonk, NY) software. The distribution of all analyzed parameters was confirmed using the Kolmogorov-Smirnov test. The χ^2^ test was used for categorical data. Spearman's ρ was used for correlation analysis. Continuous variables were tested for normal distribution using the Shapiro–Wilk test. Results for normally distributed continuous variables were expressed as means ± standard deviations. Statistical significance of the differences between means was determined by Student *t* test or analysis of variance (ANOVA), followed by post-hoc multiple comparisons using the Tukey honest significant difference (HSD) test. Correlations among continuous variables were assessed using Spearman rank correlation coefficient (*r*). Categorical variables were expressed as numbers (percentages) and were compared using Fisher exact test. Receiver operating characteristic (ROC) analysis was used to determine the separation power of the parameters. As a result of the ROC analysis, cut-off points were determined by using the Youden Index. The risk of having the values above the cut-off value was determined by performing risk analysis and the OR (odds ratio) values were obtained. Since small numbers increase the estimation bias, the Haldane correction was used. All *P*-values <.05 were considered statistically significant.

## 4 Results

The demographic features, tumor markers, and biochemical parameter levels of all subjects included in the study are shown in Table 1. No statistically significant difference was found between the groups in terms of age. The patients with CC (17 female and 18 male) and the control group (23 female and 22 male) were gender matched, so they showed no statistically significant gender difference. However, since all BC patients (45 female) were women, a statistically significant gender difference was found compared to the control group and the CC group. In BC patients, CA-15.3 levels were significantly higher than in either the patients with CC or the controls (*P* < .001 for both). CEA levels were also higher in patients with BC than in the controls (*P* < .001), and inpatients with CC compared to the controls (*P* < .001). The value of CA-19.9 was significantly higher in patients with CC compared to the controls and significantly lower in patients with BC patients than in patients with CC (*P* < .001). In patients with BC, the body mass index (BMI) was significantly higher than in the controls (*P* < .05), but no significant difference was found between the other groups. Comparison of the routine biochemical parameters of patients with CC revealed significantly lower levels of total protein, albumin, and HDL (respectively, *P* < .05, *P* < .001 and *P* < .001) and significantly higher levels of cholesterol, triglyceride and LDL (respectively, *P* < .01, *P* < .001, and *P* < .001) than in the controls. Comparison of these parameters in patients with BC revealed significantly higher levels only for LDL (*P* < .001), while total protein, albumin, and HDL were significantly lower (respectively, *P* < .001, *P* < .01, and *P* < .001) than in the controls. Comparison of the patients with BC and with CC revealed significantly lower cholesterol, triglyceride and LDL levels in the patients with BC (respectively, *P* < .05, *P* < .001, and *P* < .05).

**Table 1:** Demographic features. Tumor markers and biochemical parameters levels of all subjects (mean± SD).

	Control (n:35)	Colon cancer (n:45)	Breast cancer (n:45)	*P*		
	Mean ± SD	Mean ± S. D.	Mean ± S. D.	*P*_1_	*P*_2_	*P*_3_
Age	49.06 ± 4.84	49.88 ± 7.30	49.20 ± 7.44	1.000	1.000	1.000
Gender (F/M)	17/18	23/22	45/0	1.000	.000	.000
CA-15.3 (U / ml)	10.91 ± 3.07	14.92 ± 5.23	34.54 ± 15.25	.229	.000	.000
CA-19.9 (U / ml)	4.48 ± 2.06	27.91 ± 8.50	7.51 ± 4.14	.000	.069	.000
CEA (ng/ml)	1.50 ± 0.60	6.06 ± 7.23	5.83 ± 4.27	.000	.001	1.000
BMI (kg/m^2^)	23.47 ± 1.75	24.57 ± 3.93	25.40 ± 2.98	.372	.023	.684
Total Protein	7.28 ± 0.55	6.71 ± 1.02	6.55 ± 0.91	.017	.001	1.000
Albumin	4.05 ± 0.42	3.34 ± 0.88	3.48 ± 0.69	.000	.002	1.000
Cholesterol	179.92 ± 20.17	206.95 ± 58.57	185.45 ± 18.73	.008	1.000	.038
Triglyceride	72.55 ± 19.44	111.64 ± 37.40	86.32 ± 8.30	.000	.057	.000
HDL	55.10 ± 7.22	49.24 ± 7.25	47.98 ± 3.88	.000	.000	1.000
LDL	65.32 ± 17.87	136.63 ± 43.50	119.25 ± 16.58	.000	.000	.027
PCO (nmol/mg.protein)	0.63 ± 0.11	1.04 ± 0.16	0.99 ± 0.18	.000	.000	.370
MDA (nmol/ml)	2.63 ± 0.52	4.29 ± 0.69	3.72 ± 0.81	.000	.000	.001
AOPP (μM chloramine T)	76.94 ± 25.70	116.52 ± 25.46	104.18 ± 28.07	.000	.000	.118
NOx (μmol/L)	13.89 ± 3.58	25.55 ± 6.52	21.02 ± 6.51	.000	.000	.002
PAB (AU)	123.80 ± 21.70	159.16 ± 32.73	142.30 ± 35.50	.000	.033	.049
FRAP (mM uric acid)	14.70 ± 2.16	10.61 ± 1.78	10.47 ± 2.13	.000	.000	1.000
IMA (ng/ml)	452.05 ± 61.05	559.21 ± 140.03	527.85 ± 131.02	.000	.019	.712

AOPP = advanced protein oxidation products, BMI = body mass index, CA-15.3 = Cancer antigen 15.3, CA-19.9 = cancer antigen 19.9, CEA = carcinoembryonic antigen, FRAP = ferric reducing of antioxidant power, HDL = high density lipoprotein, IMA = ischemia modified albumin, LDL = low density lipoprotein, MDA = malondialdehyde, NOx = total nitric oxide, PAB = proxidan-antioxidant balance, PCO = protein carbonyl. P_1_, control vs colon cancer; P_2_, control vs breast cancer; P_3_, colon cancer vs breast cancer.

The PCO, MDA AOPP NOx, PAB, and IMA levels were all significantly higher in patients with CC than in the controls (for all, *P* < .001). The PCO, MDA, AOPP, NOx, PAB, and IMA values were also significantly higher in the patients with BC than in the controls (for PCO, MDA AOPP, and NOx *P* < .001; for PAB and IMA, *P* < .05). The MDA, NOx, and PAB levels were significantly lower in the BC group than in the CC group (respectively *P* < .001, *P* < .01, *P* < .05). The FRAP values were significantly lower in both the CC group and BC group than in the controls (for both *P* < .001).

Table 2 summarizes the clinicopathological features of the patients with CC and with BC. Tables 3 and 4 show the correlations between the assayed parameters and the clinicopathological data, such as the TNM stage, tumor size, presence of metastases, and the presence of receptors, in the patients with CC (Table 3) and with BC (Table 4). In the CC group, we observed moderate positive correlations between the TNM stage and CA-19.9 (*r* = 0.397, *P* = .011) and between NOx and tumor size (*r* = 0.416 *P* = .008). A moderate negative correlation was determined between PAB and CA-19.9 (*r* = −0.432 *P* = .005). IMA showed a moderate positive correlation with CA-19.9 (*r* = 0.423 *P* = .007) and a strong positive correlation with tumor size (*r* = 0.609, *P* < .001), as well as very strong correlations with TNM stage and the presence of metastasis (respectively, *r* = 0.814, *P* < .001 and *r* = 0.709, *P* < .001) (Table 3). The only parameter associated with metastasis was IMA.

**Table 2:** Clinicopathological features of the patient with colon and breast cancer.

Colon cancer		Breast cancer	
variables	n (%)	Variables	n (%)
No of patients	45 (100)	No of patients	45 (100)
TNM Stage		Grade	
*I / II / III / IV*	10 (25)/13 (32.5)/8 (20)/9 (22.5)	*1/2/3/4*	2 (5) / 20 (50) / 14 (35) / 4 (10)
Tumor size		ER	
*≤ 4* cm	21 (52.5)	−/+	15 (37.5) / 25 (62.5)
*> 4* cm	19 (47.5)		
Metastasis status		PR	
*No*	25 (62.5)	−/+	21 (52.5) / 19 (47.5)
*Yes*	15 (37.5)		
		HER2	
		−/+	29 (72.5)/ 11 (27.5)
		Classification	
		*Luminal*	11 (27.5)
		*HER2+*	18 (45)
		*Triple –*	2 (5)
		*Triple +*	9 (22.5)
		Metastasis status	
		*No*	23 (57.5)
		*Yes*	17 (42.5)

ER = estrogen receptor, PR = progesterone receptor, HER2 = Her-2/neu, TNM stage = The TNM Classification of Malignant Tumors

**Table 3:** Correlation data between tumor markers, clinicopathological data, and biochemical parameters in colon cancer patients.

		CA-15.3	CA-19.9	CEA	PCO	MDA	AOPP	NOx	PAB	FRAP	IMA
CA-15.3	*r*	–	−0.170	0.049	0.032	0.280	−0.079	−0.097	−0.175	−0.119	0.039
	p	–	0.296	0.762	0.843	0.080	0.629	0.552	0.280	0.464	0.812
CA-19.9	*r*	−0.170	–	**0.410**^**∗∗**^	−0.003	0.087	0.261	−0.051	**−0.432**^**∗∗**^	0.099	**0.423**^**∗∗**^
	p	0.296	–	**0.009**	0.984	0.595	0.104	0.756	**0.005**	0.545	**0.007**
CEA	*r*	0.049	**0.410**^**∗∗**^	–	−0.023	0.048	0.007	0.097	−0.134	0.008	0.004
	p	0.762	**0.009**	–	0.889	0.770	0.964	0.553	0.410	0.963	0.982
PCO	*r*	0.032	−0.003	−0.023	–	−0.034	0.029	−0.101	−0.120	−0.091	0.191
	p	0.843	0.984	0.889	–	0.833	0.861	0.536	0.459	0.577	0.238
MDA	*r*	0.280	0.087	0.048	−0.034	–	0.079	−0.281	−0.098	−0.153	0.221
	p	0.080	0.595	0.770	0.833	–	0.629	0.079	0.546	0.345	0.171
AOPP	*r*	−0.079	0.261	0.007	0.029	0.079	–	0.023	−0.296	0.148	0.130
	p	0.629	0.104	0.964	0.861	0.629	–	0.889	0.063	0.362	0.423
NOx	*r*	−0.097	−0.051	0.097	−0.101	−0.281	0.023	–	0.101	−0.056	−0.003
	p	0.552	0.756	0.553	0.536	0.079	0.889	–	0.537	0.731	0.986
PAB	*r*	−0.175	**−0.432**^**∗∗**^	−0.134	−0.120	−0.098	−0.296	0.101	–	0.072	−0.138
	p	0.280	**0.005**	0.410	0.459	0.546	0.063	0.537	–	0.660	0.395
FRAP	*r*	−0.119	0.099	0.008	−0.091	−0.153	0.148	−0.056	0.072	–	−0.174
	p	0.464	0.545	0.963	0.577	0.345	0.362	0.731	0.660	–	0.282
IMA	*r*	0.039	**0.423**^**∗∗**^	0.004	0.191	0.221	0.130	−0.003	−0.138	−0.174	–
	p	0.812	**0.007**	0.982	0.238	0.171	0.423	0.986	0.395	0.282	–
TNM stage	*r*	0.200	**0.397**^**∗**^	0.158	0.306	0.138	0.072	−0.123	−0.263	−0.118	**0.814**^**∗∗∗**^
	p	0.217	**0.011**	0.329	0.055	0.395	0.660	0.448	0.101	0.469	**0.000**
Tumor Size	*r*	0.020	0.310	0.223	−0.121	0.016	0.019	**0.416**^**∗∗**^	−0.176	−0.263	**0.609**^**∗∗∗**^
	p	0.902	0.051	0.166	0.456	0.922	0.906	**0.008**	0.276	0.102	**0.000**
Met	*r*	0.167	0.144	−0.026	0.276	0.197	0.095	−0.073	−0.234	−0.297	**0.709**^**∗∗∗**^
	p	0.304	0.375	0.874	0.085	0.224	0.561	0.655	0.146	0.062	**0.000**

AOPP = Advanced protein oxidation products, CA-15.3 = Cancer antigen 15.3, CA-19.9 = Cancer antigen 19.9, CEA = Carcinoembryonic antigen, FRAP = Ferric reducing of antioxidant power, IMA = Ischemia modified albumin, MDA = Malondialdehyde, Met = Metastasis, NOx = total nitric oxide, PAB = Proxidan-antioxidant balance, PCO = Protein carbonyl, TNM stage = The TNM Classification of Malignant Tumors, TNM stage = The TNM Classification of Malignant Tumors. *r* means Spearman's rank correlation coefficient and p value represents the probability value. Bold means statistically significant. ^∗^<0.05, ^∗∗^<0.01, ^∗∗∗^≤0.001.

**Table 4:** Correlation data between tumor markers, clinicopathological data and biochemical parameters in breast cancer patients.

		CA-15.3	CA-19.9	CEA	PCO	MDA	AOPP	NOx	PAB	FRAP	IMA
CA-15.3	*r*	–	−0.011	−0.009	0.036	0.081	−0.167	−0.171	0.307	−0.165	0.144
	p	–	0.944	0.957	0.826	0.620	0.304	0.290	0.054	0.309	0.377
CA-19.9	*r*	−0.011	–	−0.155	−0.049	**0.440**^**∗∗**^	−0.049	−0.210	−0.107	0.165	**0.567**^**∗∗∗**^
	p	0.944	–	0.341	0.763	**0.005**	0.763	0.192	0.509	0.308	**0.000**
CEA	*r*	−0.009	−0.155	–	0.077	0.238	−0.147	−0.086	0.057	−0.179	**0.540**^**∗∗∗**^
	p	0.957	0.341	–	0.637	0.079	0.365	0.597	0.728	0.268	**0.000**
PCO	*r*	0.036	−0.049	0.077	–	0.031	−0.082	−0.147	0.058	−0.099	0.186
	p	0.826	0.763	0.637	–	0.820	0.615	0.365	0.721	0.543	0.250
MDA	*r*	0.081	**0.440**^**∗∗**^	−0.281	0.031	–	0.086	0.198	0.050	−0.081	**0.590**^**∗∗∗**^
	p	0.620	**0.005**	0.079	0.820	–	0.238	0.463	0.884	0.686	**0.000**
AOPP	*r*	−0.167	−0.049	−0.147	−0.082	0.086	–	0.013	−0.245	0.023	−0.228
	p	0.304	0.763	0.365	0.615	0.238	–	0.935	0.128	0.889	0.158
NOx	*r*	−0.171	−0.210	−0.086	−0.147	0.198	0.013	–	0.058	−0.141	0.004
	p	0.290	0.192	0.597	0.365	0.463	0.935	–	0.721	0.386	0.979
PAB	*r*	0.307	−0.107	0.057	0.058	0.050	−0.245	0.058	–	−0.507^∗∗∗^	0.069
	p	0.054	0.509	0.728	0.721	0.884	0.128	0.721	–	0.001	0.671
FRAP	*r*	−0.165	0.165	−0.179	−0.099	−0.081	0.023	−0.141	−0**.507**^**∗∗∗**^	–	−0.118
	p	0.309	0.308	0.268	0.543	0.686	0.889	0.386	**0.001**	–	0.469
IMA	*r*	0.144	**−0.567**^**∗∗∗**^	**0.540**^**∗∗∗**^	0.186	**0.590**^**∗∗∗**^	−0.228	0.004	0.069	−0.118	–
	p	0.377	**0.000**	**0.000**	0.250	**0.000**	0.158	0.979	0.671	0.469	–
Met	*r*	0.045	**−0.666**^**∗∗∗**^	**0.234**	0.061	0.244	−0.240	0.156	0.028	0.007	0.740^∗∗∗^
	p	0.785	**0.000**	**0.146**	0.710	0.167	0.135	0.336	0.865	0.966	0.000
Grade	r	0.206	**−0.497**^**∗∗∗**^	**0.473**^**∗∗**^	0.142	0.394	−0.244	0.069	−0.017	−0.081	0.846^∗∗∗^
	p	0.202	**0.001**	**0.002**	0.383	0.069	0.130	0.672	0.917	0.621	0.000
ER	*r*	0.028	**0.584**^**∗∗∗**^	**−0.381**^**∗**^	0.024	**−0.588**^**∗∗∗**^	0.159	−0.229	−0.043	0.096	−0.735^∗∗∗^
	p	0.862	**0.000**	**0.015**	0.881	**0.000**	0.327	0.155	0.790	0.555	0.000
PR	*r*	−0.043	0.514^∗∗^	−.433^∗∗^	−0.012	−0.282	0.212	−0.081	−0.204	0.214	−0.576^∗∗∗^
	p	0.790	0.001	0.005	0.940	0.056	0.190	0.619	0.208	0.185	0.000
HER2	*r*	0.069	**−0.413**^**∗∗**^	**0.472**^**∗∗**^	0.192	**0.627**^**∗∗∗**^	−0.013	0.069	0.135	−0.158	0.734∗^∗∗^
	p	0.671	**0.008**	**0.002**	0.234	**0.000**	0.937	0.670	0.405	0.330	0.000

AOPP = Advanced protein oxidation products, CA-15.3 = cancer antigen 15.3, CA-19.9 = cancer antigen 19.9, CEA = carcinoembryonic antigen, ER = estrogen receptor, FRAP = ferric reducing of antioxidant power, HER2 = Her-2/neu receptor, IMA = ischemia modified albumin, MDA = malondialdehyde, Met = Metastasis, NOx = total nitric oxide, PAB = proxidan-antioxidant balance, PCO = protein carbonyl, PR = progesterone receptor. *r* means Spearman's rank correlation coefficient and p value represents the probability value. Bold means statistically significant. ^∗^<0.05; ^∗∗^<0.01; ^∗∗∗^≤0.001.

In the BC group, MDA showed a moderate positive correlation with CA-19.9 (*r* = 0.440, *P* = .005) and a strong negative correlation with the presence of ER (*r* = −0.588, *P* < .001), as well as a strong positive correlation with IMA (*r* = 0.590, *P* < .001) and with the presence of HER2 (*r* = 0.627, *P* < .001). A strong positive correlation was found between IMA levels and CEA (*r* = 0.540, *P* < .001), and strongly positive correlations were found for IMA and the presence of metastasis (*r* = 0.740, *P* < .001), tumor grade (*r* = 0.846, *P* < .001), and the presence of HER2 (*r* = 0.734, *P* < .001). IMA also showed a strongly negative correlation with the presence of PR (*r* = −0.576, *P* < .001) and a very strongly negative correlation with the presence of ER (*r* = −0.735, *P* < .001) (Table 4).

**Figure 1:**
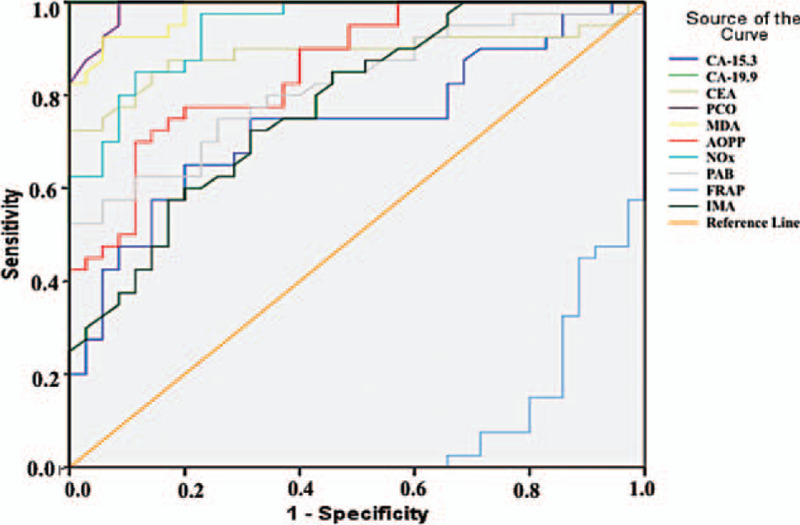
Diagnostic criteria of ROC curve for tested parameters and tumor markers between colon cancer patient and control groups.

**Figure 2:**
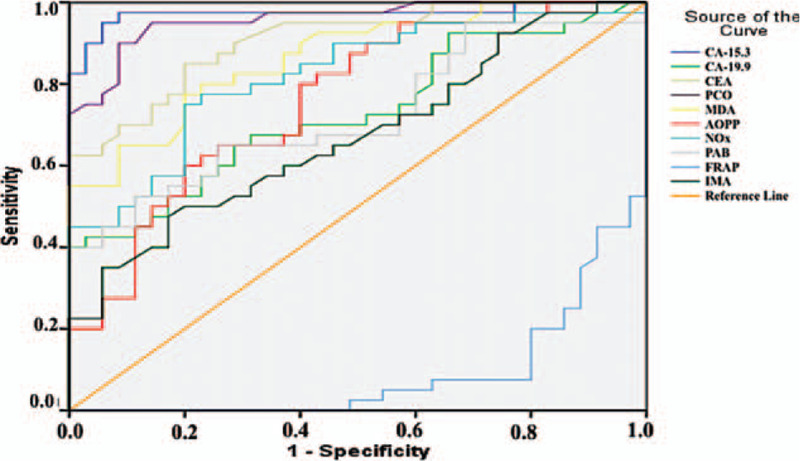
Diagnostic criteria of ROC curve for tested parameters and tumor markers between breast cancer patient and control groups.

Figure 1 and Table 5 summarize the diagnostic criteria of the ROC curve for the tested parameters and tumor markers used to differentiate CC from the control subjects. All parameters except FRAP could be used to distinguish patients with CC from the control individuals. We found that the parameter with the highest specificity and sensitivity value was CA-19.9 (for both 100%), whereas the highest specificity and sensitivity values among the parameters we examined were obtained for PCO (sensitivity: 100%, specificity: 91.4%) and MDA (sensitivity: 92.5%, specificity: 94.3%, Table 5). Similarly, for patients with BC, all parameters except FRAP were significant (Fig. 2), but the parameter with the best specificity and sensitivity among all the parameters we examined and among the tumor markers was PCO (sensitivity: 90%, specificity: 91.4%, Table 6). The higher FRAP level in control individuals indicated that FRAP could be a good marker for differentiating healthy individuals.

**Table 5:** Diagnostic criteria of ROC curve as a measure of predictive discrimination colon cancer patients from controls.

			Asymptotic 95% CI				
Variables	AUC	*P*	Lower Bound	Upper Bound	Cut-off value	Sensitivity (%)	Specificity (%)
CA-15.3	**0.737**	**.000**	0.623	0.851	12.935	65.0	20.0
CA-19.9	**1.000**	**.000**	1.000	1.000	11.065	**100**	**100**
CEA	**0.890**	**.000**	0.807	0.974	2.960	72.5	100
PCO	**0.991**	**.000**	0.977	1.000	0.755	**100**	**91.4**
MDA	**0.981**	**.000**	0.959	1.000	3.425	**92.5**	**94.3**
AOPP	**0.859**	**.000**	0.778	0.940	103.935	**70.0**	**88.6**
NOx	**0.944**	**.000**	0.898	0.990	16.335	97.5	77.1
PAB	**0.819**	**.000**	0.725	0.914	155.000	**52.5**	**100**
FRAP	**0.082**	**.000**	0.020	0.144	–	–	–
IMA	**0.778**	**.000**	0.676	0.881	466.485	72.5	68.6

The area under the receiver operating characteristic curve (ROC) was calculated as a measure of predictive discrimination of colon cancer. Bold means statistically significant. Cut-off points were determined by using the Youden Index. The cut-off value, specificity and sensitivity values were not calculated for the parameters with very low AUC values (below 0.1). AOPP = advanced protein oxidation products, AUC = area under the curve, CA-15.3 = cancer antigen 15.3, CA-19.9 = cancer antigen 19.9, CEA = Carcinoembryonic antigen, CI, confidence interval, FRAP = ferric reducing of antioxidant power, IMA = ischemia modified albumin, MDA = malondialdehyde, NOx = total nitric oxide, PAB = proxidan-antioxidant balance, PCO = protein carbonyl.

**Table 6:** Diagnostic criteria of ROC curve as a measure of predictive discrimination breast cancer patients from controls.

			Asymptotic 95% CI				
Variables	AUC	*P*	Lower Bound	Lower Bound	Cut-off value	Sensitivity (%)	Specificity (%)
CA153	**0.974**	**.000**	0.934	1.000	15.835	**95.0**	**94.3**
CA19-9	**0.729**	**.001**	0.616	0.842	9.065	40.0	**100**
CEA	**0.907**	**.000**	0.844	0.971	1.750	**85.0**	**80.0**
PCO	**0.959**	**.000**	0.919	0.999	0.755	**90.0**	**91.4**
MDA	**0.867**	**.000**	0.788	0.945	3.030	**77.5**	**80.0**
AOPP	**0.761**	**.000**	0.654	0.869	94.695	60.0	**80.0**
NOx	**0.821**	**.000**	0.727	0.915	17.040	75.0	**80.0**
PAB	**0.726**	**.001**	0.611	0.840	148.495	52.5	**88.6**
FRAP	**0.088**	**.000**	0.026	0.151	–	–	**–**
IMA	**0.674**	**.010**	0.553	0.794	481.435	47.5	**82.9**

The area under the receiver operating characteristic curve (ROC) was calculated as a measure of predictive discrimination of breast cancer. AUC = area under the curve, CI = confidence interval. Bold means statistically significant. Cut-off points were determined by using the Youden Index. The cut-off value = specificity and sensitivity values were not calculated for the parameters with very low AUC values (below 0.1).

**Figure 3:**
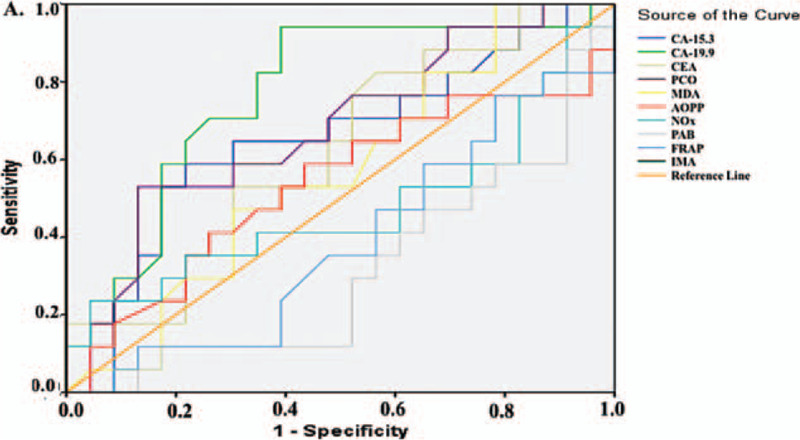
Diagnostic criteria of ROC curve for tested parameters and tumor markers in colon cancer patients A. Between high TNM stage (TNM stage 4 and 3) and low-intermediate stage (TNM stage 2 and 1).

**Figure 4:**
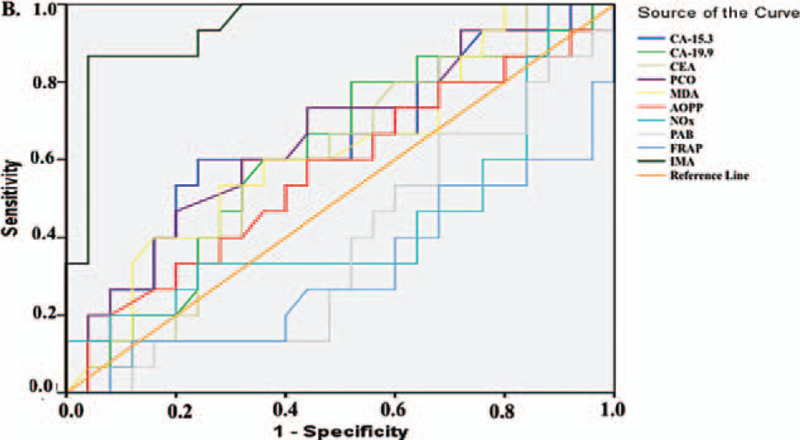
Diagnostic criteria of ROC curve for tested parameters and tumor markers in colon cancer patients B. Between metastasis and non-metastasis.

For further analysis, patients with CC were divided into 2 groups of high TNM Stage (TNM Stage III and IV) and low-intermediate TNM stage (TNM Stage I and II) according to their TNM stages and further divided into 2 groups according to the presence of metastasis. Table 7 shows the mean ± S.D. values for the tumor markers and all parameters we analyzed for these subgroups. The PAB value was significantly higher in the low-intermediate TNM stage group than in the high TNM stage group (*P* < .05), while the IMA level was significantly lower (*P* < .001). IMA was the only parameter that showed a significant difference in patients with CC separated by the presence of metastasis and the IMA level was significantly lower in patients without metastasis than with metastases (*P* < .001) (Table 7). The ROC analysis performed in these subgroups for all parameters (Figs. 3 and 4) revealed that. IMA was the best biomarker for differentiating patients with a high TNM stage from those with a low-intermediate TNM stage (Table 8; AUC: 1.000, *P* < .001, sensitivity: 100%, specificity: 100%) and for differentiating patients with metastasis from those without metastasis (Table 9, AUC: 0.943, *P* < .001, sensitivity: 86.7%, specificity: 96%).

**Table 7:** Biochemical parameters and tumor marker levels in colon cancer cases (mean± SD). Metastasis **A.** Between high TNM stage (TNM stage 4 and 3) and low-intermediate TNM stage (TNM stage 2 and 1) **B.** Between metastasis and non-metastasis.

	A. TNM Stage			B. Metastasis		
	I + II (n:23)	III +IV (17)		No (n:25)	Yes (n:15)	
	Mean ± S. D.	Mean ± S. D.	P_T_	Mean ± S. D.	Mean ± S. D.	P_M_
CA-15.3	14.04 ± 5.47	16.09 ± 4.80	0.226	14.25 ± 5.40	16.03 ± 4.90	0.304
CA.19.9	**24.58 ± 7.16**	**32.40 ± 8.26∗∗**	0.003	26.97 ± 9.12	29.47 ± 7.38	0.375
CEA	4.59 ± 2.87	8.04 ± 10.42	0.137	6.20 ± 8.90	5.82 ± 3.08	0.874
PCO	1.00 ± 0.15	1.10 ± 0.15	0.051	1.01 ± 0.15	1.10 ± 0.15	0.085
MDA	4.22 ± 0.73	4.39 ± 0.62	0.427	4.19 ± 0.69	4.46 ± 0.67	0.224
AOPP	115.60 ± 24.15	117.77 ± 27.85	0.794	114.68 ± 24.94	119.60 ± 26.89	0.561
NOx	25.67 ± 5.63	25.40 ± 7.74	0.900	25.92 ± 6.01	24.95 ± 7.47	0.655
PAB	**168.30 ± 32.44**	**146.79 ± 29.70∗**	0.038	165.02 ± 33.18	149.39 ± 30.57	0.146
FRAP	10.92 ± 1.75	10.19 ± 1.78	0.207	11.01 ± 1.61	9.93 ± 1.90	0.062
IMA	**465.74 ± 23.65**	**685.67 ± 132.19∗∗∗**	0.000	**483.25 ± 68.54**	**685.80 ± 138.18∗∗∗**	0.000

AOPP = advanced protein oxidation products, CA-15.3 = cancer antigen 15.3, CA-19.9 = cancer antigen 19.9, CEA = carcinoembryonic antigen, FRAP = ferric reducing of antioxidant power, IMA = ischemia modified albumin, MDA = malondialdehyde, NOx = total nitric oxide, PAB = proxidan-antioxidant balance, PCO = protein carbonyl. Bold means statistically significant. **The P**_**T**_** value** shows the statistical significance between high TNM stage (TNM stage 4 and 3) and low-intermediate TNM stage (TNM stage 2 and 1), **the P**_**M**_** value** indicates the statistical significance between patients with metastasis (Yes) and without (No) metastasis in colon cancers cases.

**Table 8:** Diagnostic criteria of ROC curve as a measure of predictive discrimination colon cancer patients with high TNM stage (TNM stage 4 and 3) from low-intermediate stage (TNM stage 2 and 1).

According to TNM stage							
			Asymptotic 95% CI				
Variables	AUC	*P*	Lower Bound	Upper Bound	Cut-off value	Sensitivity (%)	Specificity (%)
CA-15.3	0.651	.106	0.473	0.829	15.650	58.8	78.3
CA-19.9	**0.767**	**.004**	**0.614**	**0.921**	**25.695**	**94.1**	**60.9**
CEA	0.625	.180	0.450	0.800	3.375	82.4	43.5
PCO	0.680	.054	0.511	0.850	1.135	52.9	87.0
MDA	0.565	.485	0.386	0.744	3.445	100	21.7
AOPP	0.536	.702	0.345	0.726	111.355	58.8	56.5
NOx	0.473	.774	0.277	0.669	33.470	23.5	95.7
PAB	**0.309**	**.042**	**0.142**	**0.477**	**154.365**	**41.2**	**39.1**
FRAP	0.384	.213	0.207	0.561	–	–	–
IMA	**1.000**	**.000**	**1.000**	**1.000**	**512.335**	**100**	**100**

The area under the receiver operating characteristic curve (ROC) was calculated as a measure of predictive discrimination of high TNM stage in colon cancer. Bold means statistically significant. Cut-off points were determined by using the Youden Index. The cut-off value = specificity and sensitivity values were not calculated for the parameters with low AUC values (below 0.4). AOPP = advanced protein oxidation products, AUC = area under the curve, CA-15.3 = cancer antigen 15.3, CA-19.9 = cancer antigen 19.9, CEA = carcinoembryonic antigen, CI = confidence interval, FRAP = ferric reducing of antioxidant power, IMA = ischemia modified albumin, MDA = malondialdehyde, NOx = total nitric oxide, PAB = proxidan-antioxidant balance, PCO = protein carbonyl.

**Table 9:** Diagnostic criteria of ROC curve as a measure of predictive discrimination colon cancer patients with metastasis from without metastasis.

According to metastasis status							
			Asymptotic 95% CI				
Variables	AUC	*P*	Lower Bound	Upper Bound	Cut-off value	Sensitivity (%)	Specificity (%)
CA-15.3	0.639	.146	0.457	0.821	15.650	60.0	76.0
CA-19.9	0.611	.246	0.429	0.792	25.695	80.0	48.0
CEA	0.601	.288	0.422	0.780	5.675	60.0	68.0
PCO	0.657	.099	0.481	0.834	1.016	73.3	56.0
MDA	0.619	.214	0.438	0.800	4.620	53.3	72.0
AOPP	0.567	.485	0.377	0.756	111.355	60.0	56.0
NOx	0.437	.511	0.237	0.637	35.215	13.3	100
PAB	0.379	.204	0.201	0.557	–	–	–
FRAP	0.313	.051	0.134	0.492	12.575	13.3	88.0
IMA	**0.943**	**.000**	**0.872**	**1.000**	**553.040**	**86.7**	**96.0**

The area under the receiver operating characteristic curve (ROC) was calculated as a measure of predictive discrimination of metastasis in colon cancer. AUC = area under the curve, CI = confidence interval. Bold means statistically significant. Cut-off points were determined by using the Youden Index. The cut-off value, specificity and sensitivity values were not calculated for the parameters with low AUC values (below 0.4). AOPP = advanced protein oxidation products, CA-15.3 = cancer antigen 15.3, CA-19.9 = cancer antigen 19.9, CEA = carcinoembryonic antigen, FRAP = ferric reducing of antioxidant power, IMA = ischemia modified albumin, MDA = malondialdehyde, NOx = total nitric oxide, PAB = proxidan-antioxidant balance, PCO = protein carbonyl.

**Figure 5:**
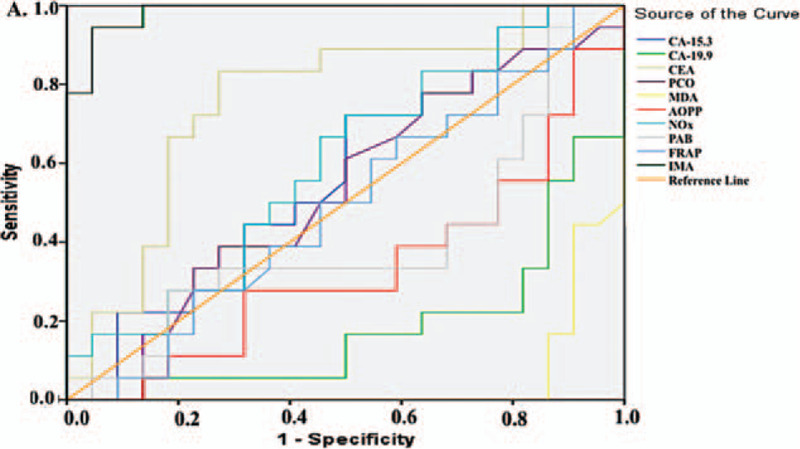
Diagnostic criteria of ROC curve for tested parameters and tumor markers in breast cancer patients A. Between high-grade (grade 4 and grade 3) and low-intermediate grade (grade 2 and grade 1).

**Figure 6:**
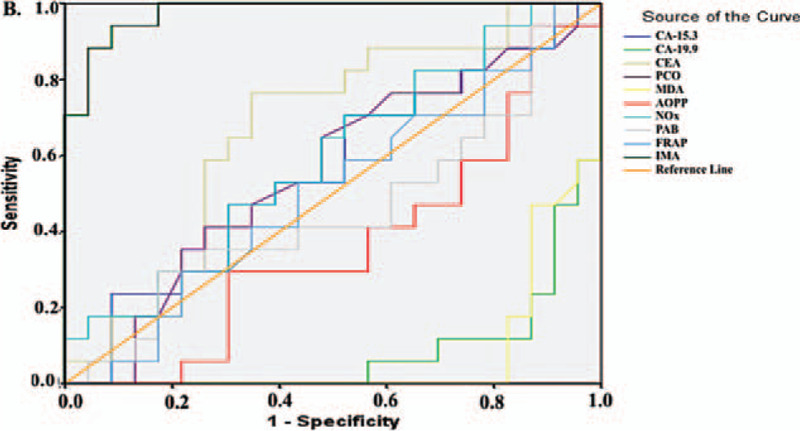
Diagnostic criteria of ROC curve for tested parameters *and* tumor markers in breast cancer patients B. Between metastasis and non-metastasis.

Similarly, patients with BC were divided into 2 subgroups of high and low-medium grade disease and further divided into 2 subgroups according to the presence of metastases. The MDA levels were significantly lower in the high-grade subgroup (*P* < .01), whereas CEA and IMA levels were significantly higher (for both *P* < .001). In addition, the subgroup with metastasis showed significantly lower MDA levels and significantly higher IMA levels (*P* < .001 for both) (Table 10). The ROC analyses, performed separately for subgroups according to grade (Fig. 5 and Table 11) and metastasis (Fig. 6 and Table 12), revealed results similar to those obtained for the CC subgroups. IMA was the most appropriate parameter among the parameters we studied for detecting detect high grade (Table 11, AUC: 0.985, *P* < .001; sensitivity: 94.40%, specificity: 95.50%) and metastasis (Table 12, AUC: 0.977, *P* < .001; sensitivity: 94.12%, specificity: 91.30%).

**Table 10:** Biochemical parameters and tumor marker levels in breast cancer cases (mean± SD). A. Between high-grade (grade 4 and grade 3) and low-intermediate grade (grade 2 and grade 1; B. Between metastasis and non-metastasis.

	Grade			Metastasis		
	1 + 2 (n:22)	3 + 4 (n:18)		No (n:23)	Yes (n:17)	
	Mean ± S. D.	Mean ± S. D.	P_G_	Mean ± S. D.	Mean ± S. D.	P_M_
CA-15.3	33.79 ± 17.65	35.45 ± 12.14	0.477	33.96 ± 17.11	35.32 ± 12.79	0.785
CA-19.9	**9.41 ± 4.00**	**5.19 ± 3.02∗∗∗**	**0.000**	**9.85 ± 3.72**	**4.35 ± 2.05∗∗∗**	**0.000**
CEA	**4.20 ± 3.77**	**7.82 ± 4.08∗∗**	**0.007**	4.98 ± 4.35	6.97 ± 3.99	0.146
PCO	0.99 ± 0.19	0.99 ± 0.17	0.402	0.98 ± 0.19	1.00 ± 0.18	0.710
MDA	**4.28 ± 0.59**	**3.03 ± 0.40∗∗∗**	**0.000**	**4.22 ± 0.64**	**3.04 ± 0.41∗∗∗**	**0.000**
AOPP	111.33 ± 29.41	95.44 ± 24.32	0.058	109.91 ± 31.89	96.44 ± 20.27	0.135
NOx	20.00 ± 6.76	22.28 ± 6.13	0.129	20.16 ± 6.69	22.19 ± 6.26	0.336
PAB	148.40 ± 42.15	140.96 ± 26.34	0.452	141.46 ± 41.45	143.43 ± 26.59	0.865
FRAP	10.49 ± 2.27	10.46 ± 2.00	0.424	10.46 ± 2.22	10.49 ± 2.05	0.966
IMA	**441.22 ± 34.10**	**633.74 ± 127.91∗∗∗**	**0.000**	**445.53 ± 41.07**	**639.23 ± 128.85∗∗∗**	**0.000**

AOPP = advanced protein oxidation products, CA-15.3 = cancer antigen 15.3, CA-19.9 = cancer antigen 19.9, CEA = carcinoembryonic antigen, FRAP = ferric reducing of antioxidant power, IMA = ischemia modified albumin, MDA = malondialdehyde, NOx = total nitric oxide, PAB = proxidan-antioxidant balance, PCO = protein carbonyl. Bold means statistically significant. **The P**_**G**_** value** shows the statistical significance between low-intermediate grade (grade 1+2) and high-grade (grade 3+4) groups. **The P**_**M**_** value** indicates the statistical significance between breast cancer patients with metastasis (Yes) and without (No) metastasis.

**Table 11:** Diagnostic criteria of ROC curve as a measure of predictive discrimination breast cancer patients with high-grade (grade 4 and grade 3) from low-intermediate grade (grade 2 and grade 1).

According to grade							
			Asymptotic 95% CI				
Variables	AUC	*P*	Lower Bound	Upper Bound	Cut-off value	Sensitivity (%)	Specificity (%)
CA-15.3	0.572	.438	0.393	0.751	28.225	72.22	50.00
CA-19.9	0.184	.001	0.049	0.320	-	-	-
CEA	0.760	.005	0.604	0.916	3.760	**83.33**	72.73
PCO	0.532	.734	0.350	0.714	0.875	77.78	36.36
MDA	0.049	.000	0.000	0.113	–	–	–
AOPP	0.333	.073	0.162	0.505	–	–	–
NOx	0.598	.289	0.422	0.775	17.835	72.22	50.00
PAB	0.407	.314	0.220	0.593	164.015	27.78	81.82
FRAP	0.494	.946	0.312	0.675	10.325	50.00	54.55
IMA	**0.985**	**.000**	**0.957**	**1.000**	**488.905**	**94.40**	**95.50**

The area under the receiver operating characteristic curve (ROC) was calculated as a measure of predictive discrimination of high tumor grade in breast cancer. Bold means statistically significant. Cut-off points were determined by using the Youden Index. The cut-off value = specificity and sensitivity values were not calculated for the parameters with low AUC values (below 0.4). AOPP = advanced protein oxidation products, AUC = area under the curve, CA-15.3 = Cancer antigen 15.3, CA-19.9 = cancer antigen 19.9, CEA = carcinoembryonic antigen, CI = confidence interval, FRAP = ferric reducing of antioxidant power, IMA = ischemia modified albumin, MDA = malondialdehyde, NOx = total nitric oxide, PAB = proxidan-antioxidant balance, PCO = protein carbonyl.

**Table 12:** Diagnostic criteria of ROC curve as a measure of predictive discrimination breast cancer patients with metastasis from without metastasis.

According to metastasis status							
			Asymptotic 95% CI				
Variables	AUC	*P*	Lower Bound	Upper Bound	Cut-off value	Sensitivity (%)	Specificity (%)
CA-15.3	0.560	.520	0.378	0.743	28.23	70.59	47.83
CA-19.9	0.084	.000	0.000	0.170	–	–	–
CEA	0.668	.073	0.496	0.839	3.76	76.47	65.22
PCO	0.556	.547	0.373	0.739	0.97	64.71	52.17
MDA	0.075	.000	0.000	0.157	–	–	–
AOPP	0.366	.151	0.191	0.540	77.01	**94.12**	13.04
NOx	0.593	.318	0.415	0.772	17.84	70.59	47.83
PAB	0.458	.652	0.271	0.645	164.02	29.41	82.61
FRAP	0.504	.967	0.321	0.687	10.33	52.94	56.52
IMA	**0.977**	**.000**	**0.941**	**1.000**	**488.91**	**94.12**	**91.30**

The area under the receiver operating characteristic curve (ROC) was calculated as a measure of predictive discrimination of metastasis in breast cancer. Bold means statistically significant. Cut-off points were determined by using the Youden Index. The cut-off value, specificity and sensitivity values were not calculated for the parameters with very low AUC values (below 0.1). AOPP = advanced protein oxidation products, AUC = area under the curve, CA-15.3 = cancer antigen 15.3, CA-19.9 = cancer antigen 19.9, CEA = Carcinoembryonic antigen, CI = confidence interval, FRAP = ferric reducing of antioxidant power, IMA = ischemia modified albumin, MDA = malondialdehyde, NOx = total nitric oxide, PAB = proxidan-antioxidant balance, PCO = protein carbonyl.

## 5 Discussion

The available evidence now strongly supports an involvement of ROS and RNS in both the onset and increase in multi-stage carcinogenesis.^[22]^ In the current study, we found significantly higher levels of PCO, AOPP, MDA, NO, IMA, and PAB and significantly lower FRAP levels in patients with CC or BC than in the control group. The IMA levels were also positively correlated with TNM stage, tumor size, and metastasis in patients with either CC or BC. Moreover, the IMA levels were positively correlated with the MDA levels and with HER2 in patients with BC. Taken together, these data show that IMA can be especially associated with lipid peroxidation and poor prognosis in patients with BC and especially associated with PAB and aggressive and high-grade disease in patients with CC. Consistent with the literature, these conditions are thought to be due to ischemia and secondary oxidative stress in cancer pathogenesis.

ROS at high levels attack proteins, lipids, and DNA, thereby damaging the cell through protein oxidation, lipid peroxidation, and DNA damage. AOPP is defined as cross-linked protein products containing dityrosine and is considered a reliable marker for detecting protein damage.^[23,24]^ The interaction of ROS with proteins results in the formation of PCO products from many amino acid residues, such as histidine, proline, arginine, and lysine, that form the protein backbone.^[25]^ Increased AOPPs values are seen in various cancers, including CC and BC.^[26–36]^ In the current study, increased protein oxidation in the patients with CC or BC was also confirmed using a novel method (the AOPP and PCO assays) that provides information regarding the degree of oxidative damage to proteins. The PCO assay had high sensitivity and specificity for differentiating patients with CC and BC from the controls, whereas the AOPP assay showed only high specificity.

Both carbonyl stress and protein oxidation may contribute to the progression of CC and BC. For example, Tesarová et al^[32]^ showed that patients with BC had an early increase in AGEs (a marker of the carbonyl stress), followed by a further increase of AGEs and elevation of AOPP (a marker of oxidative stress) in patients with progressive disease. Similarly, Kilic et al^[30]^ found significantly higher AOPP levels in patients with BC than in controls, and they suggested that BC and oxidative stress are closely related. Another study reported significantly higher values of PCO and AOPP in patients with gastric cancer (GC) than in controls.^[34]^ Increased PCO has also been found in patients with colorectal cancer (CRC); for example, Chang et al^[33]^ found increased levels of AOPP and PCO, confirming the presence of oxidative stress in CRC patients. Similarly, Chandramathi et al^[31]^ showed elevated urinary AOPP in patients with BC or CRC compared to control subjects. The levels of urinary AOPP were also significantly higher in the patients with CRC than in those with BC. Urinary AOPP could therefore serve as useful non-invasive oxidative stress biomarker for CRC, whereas only AOPP levels are sufficiently sensitive to estimate oxidative damage in BC. The ability to use non-invasive diagnostic biochemical parameters would represent a very important contribution to the diagnostic arsenal for CC and BC, considering the high incidence of these deadly diseases. In this regard, AOPP and PCO levels appear to be of appreciable value, although further studies are warranted.

MDA, the end product of lipid peroxidation, can also be used to estimate the intensity of oxidative stress or damage caused by lipid peroxidation. In present study, the MDA levels were significantly higher in the patients with CC or BC compared to the control group. MDA levels were also significantly lower in the BC group than in the CC group, in agreement with a previous study that showed increased MDA levels in patients with CC.^[37]^ Rašić et al^[38]^ demonstrated a progressive increase in serum levels of MDA in patients with CRC, with the highest value reached in the fourth stage of CRC. MDA levels were significantly higher in the pT4 group than in the pT3 and pT2 groups of patients with CRC. Significantly higher levels of MDA were also found in the N1 and N2 groups than in the N0 group of patients with CRC, as well as in patients with metastatic disease than in those without metastasis. Kilic et al^[30]^ found significantly higher MDA levels in patients with BC than in controls. Chandramathi et al^[31]^ also showed elevated urinary MDA levels in patients with CRC, in agreement with another study that showed elevated plasma MDA levels in patients with CRC.^[38]^

Nitric oxide (NO) is a relatively stable free-radical gas that readily diffuses across cell membranes and into cells, where it reacts with molecular targets. NO, as a free radical, is a highly reactive molecule within biological systems and is capable of interacting with other free radicals, molecular oxygen, and heavy metals. The biological effects of NO can be mediated by the products of different NO metabolites. Initial findings have suggested that the NO generated by immune cells is cytostatic or cytotoxic for tumor cells; however, more recent findings have shown that NO can also show apparently contradictory activity and promote tumor growth.^[39]^ The data in the present study indicate that NOx and PAB are present at significantly higher levels in patients with BC than in healthy controls, but these levels are significantly lower in the BC group when compared to the CC group. These data may explain the success of small clinical trials and the failure of larger clinical trials; since excess amounts of vitamins may create oxidative stress and have an adverse effect on PAB.^[40]^

Recent studies have shown that dietary TAC (D-TAC) may affect risk of cancer; however, the findings are conflicting. In the present study, the FRAP values were significantly lower in both the CC group and BC group compared to control, and FRAP is more accurate than other methods for estimating of D-TAC. Halvorsen et al^[41]^ observed an inverse association between D-TAC and risk of CRC. Similarly, a meta-analysis indicated that adherence to a low-fat diet with high content of antioxidants was associated with better prognosis in patients with BC.^[42]^ TAC provides an adequate and efficient protection against the oxidative stress that can result in protein and lipid damage.

Ischemia-modified albumin (IMA) is an altered type of serum albumin generated by ROS^[43,44]^ and is accepted as a reliable biomarker of oxidative stress.^[13]^ Elevated serum IMA is demonstrated in various diseases associated with inflammation and oxidative stress, and serum IMA level has been shown to increase in BC and CC.^[8,10–16]^ Satoh et al^[16]^ proposed that serum IMA measurement can serve as a marker for monitoring the postoperative course in patients following colorectal surgery, but their study had limitations (i.e., small samples, no comparisons with controls and other diseased cases), and further research is warranted to confirm their prediction. In the present study, IMA levels were significantly lower in the low-intermediate TNM stage group than in high TNM stage group. IMA was also the only parameter that showed a significant difference in patients with metastatic CC, as it was significantly lower in patients without metastasis than with metastases. IMA was the most appropriate of the parameters studied here for detection of high grade disease and metastasis. Ellidag et al^[8]^ found that the oxidative/antioxidant status was impaired in favor of oxidative stress in patients with CRC, but this observation was not confirmed by IMA measurements. Further studies are needed to verify the relationship between IMA and oxidative stress parameters in CRC and other cancers.

The present study had some limitations. One was its relatively small sample size, which may limit the generalizability of the results. Another limitation was that we were unable to adjust for other oxidative stress markers. A third limitation was the lack of data in term of patients’ diet and nutritional status, as the circulating levels of oxidative stress factors could be affected by a patient's diet. A further limitation is that patients with solid tumors other than BC and CC were not included in the study. In addition, since our patients did not have a long-term follow-up, no comment can be made regarding surveillance or disease prognosis. A last limitation is our study's observational design. Therefore, further mechanistic explorations are needed to verify the present findings.

## 6 Conclusions

Oxidative stress occurs in various cancers, as evidenced by the increased circulation of oxi and nitro radicals and a general weakening of cellular redox homeostasis, and these changes cause tumorigenesis. In the present study, PAB was elevated in the serum of patients with either BC or CC. Increases in oxidative status indicate a reduction in potential antioxidant defenses, and disruption of this balance probably plays a role in cancer pathogenesis. IMA appears to be a reliable biomarker of this oxidative stress and may reflect tumor ischemia, which leads to proinflammatory reaction cascades and enhanced ROS production. Patients with BC and CC had impaired oxidative/antioxidant ratios that favored oxidative stress. The ROC analysis showing IMA sensitivity above 80% supports the use of IMA as an auxiliary biomarker in diagnosis. For this reason, we believe that detailed studies in which the steps of carcinogenesis are examined one by one in terms of oxidative stress and antioxidant activity can confirm this possibility.

## Acknowledgments

The authors would like to thank all participants in this study.

## Author contributions

**Conceptualization:** Berrin Papila, Volkan Sozer, Sinem Durmus, Pinar Cigdem Kocael, Fatih Orkun Kundaktepe, Cigdem Papila, Remise Gelisgen, Hafize Uzun.

**Data curation:** Berrin Papila, Pinar Cigdem Kocael, Cigdem Papila, Remise Gelisgen, Hafize Uzun.

**Formal analysis:** Volkan Sozer, Sinem Durmus, Cigdem Papila, Remise Gelisgen, Hafize Uzun.

**Funding acquisition:** Berrin Papila, Fatih Orkun Kundaktepe, Cigdem Papila, Hafize Uzun.

**Investigation:** Berrin Papila, Sinem Durmus, Pinar Cigdem Kocael, Fatih Orkun Kundaktepe, Cigdem Papila, Remise Gelisgen, Hafize Uzun.

**Methodology:** Berrin Papila, Volkan Sozer, Sinem Durmus, Cigdem Papila, Remise Gelisgen, Hafize Uzun.

**Project administration:** Berrin Papila, Pinar Cigdem Kocael, Cigdem Papila, Hafize Uzun.

**Resources:** Berrin Papila, Hafize Uzun.

**Software:** Berrin Papila, Sinem Durmus, Hafize Uzun.

**Supervision:** Berrin Papila, Hafize Uzun.

**Validation:** Berrin Papila, Sinem Durmus, Cigdem Papila, Hafize Uzun.

**Visualization:** Berrin Papila, Cigdem Papila, Hafize Uzun.

**Writing – original draft:** Berrin Papila, Volkan Sozer, Sinem Durmus, Pinar Cigdem Kocael, Fatih Orkun Kundaktepe, Cigdem Papila, Remise Gelisgen, Hafize Uzun.

**Writing – review & editing:** Berrin Papila, Volkan Sozer, Cigdem Papila, Hafize Uzun.
